# Age is associated with reduced urethral pressure and afferent activity in rat

**DOI:** 10.14814/phy2.15107

**Published:** 2021-11-09

**Authors:** Arezoo Geramipour, Zachary C. Danziger

**Affiliations:** ^1^ Department of Biomedical Engineering Florida International University Miami Florida USA

**Keywords:** aging, intraurethral pressure, neuropathy, pudendal afferents activity

## Abstract

Age‐related changes in the lower urinary tract (LUT) can affect the coordination of reflexes and increase the incidence of bladder disorders in elderly. This study examines the age‐related loss of urethral signaling capability by measuring the afferent activity directly. We find that less urethral pressure develops in response to fluid flow in old rats compared to young rats and that pressure and flow evoke less urethral afferent activation. These findings are consistent with our previous study demonstrating that the urethra‐to‐bladder reflex, which is required for efficient voiding, becomes weaker with age. We measured the pudendal afferent response in young (4–7 months) and old (18–24 months) rats to fluid flow in the urethra across a range of flow rates. We used paraffin embedding and hematoxylin and eosin staining to quantify age‐related changes in the sensory branch of the pudendal nerve. Urethral afferent signaling in response to the same urethral flow rates was weaker in older animals. That is, the sensitivity of urethra afferents to flow decreased with age, and higher flow rates were required in older animals to recruit urethra afferents. There was also a reduction in the myelin thickness of pudendal afferents in old rats, which is a possible contributing factor to the sensory activity. Furthermore, the same flow rates evoked less pressure in the urethras of old animals, indicating there is an age‐related change of the urethral tissue that reduces the pressure stimulus to which these afferents respond. These results help characterize the underlying changes in LUT system with age.

## Graphical abstract

The prevalence of underactive bladder (UAB) symptoms increases with age; however, the underlying mechanism of age‐related bladder dysfunction is unclear. In this study on rats, we found age‐related reductions of overall urethral sensory outflow, urethral pressure development during fluid flow, and myelin present in urethral afferents, suggesting urethral neuropathy plays a role in UAB.

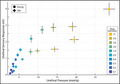

## 1 INTRODUCTION

Aging is frequently associated with lower urinary tract (LUT) symptoms (like urgency and hesitancy to void), which affects geriatric health and reduces quality of life. Recent studies have established that LUT structure changes with age, potentially increasing the incidence of bladder/bowel disorders in the elderly (Chancellor & Diokno, 2016; Tyagi et al., 2014). The mechanisms underlying these changes are likely to be multifactorial and complex. Due to the increase in the elderly population to 20% by 2050 (United Nations, Department of Economic, & Social Affairs PD, 2019), and its effect on global healthcare expenses and quality of life, a thorough understanding of age‐related LUT changes is a topic of considerable interest.

Recent studies about the effect of aging on the LUT highlighted a lack of afferent signaling or integrative control of sensory information in the spinal and supraspinal centers, resulting in weak efferent signaling (Finkbeiner, 1993; Glipin et al., 1986; Nakayama et al., 1998; Verdú et al., 2000) or compromised urethral sensation (Kenton et al., 2007). There are two likely sources from which a reduction in urethral sensory outflow might arise. First, hypotrophy of the pudendal nerve that carries urethral afferents could directly reduce pudendal afferent activity by damaging fibers that convey urethral sensation. Second, an increase in compliance of the urethra could reduce the afferent signaling during a void. As the bladder pushes fluid into the urethra, the pressure buildup increases urethral wall stress that drives afferent activation (Le Feber et al., 1998); therefore, the same amount of fluid entering a more compliant urethra generates less pressure and wall stress, and in turn, less afferent signaling.

Evidence in the literature is consistent with age‐related changes in the LUT. Degradation of urethral afferents and LUT denervation is present in older populations (Chai et al., 2000; Collas & Malone‐Lee, 1996; Finkbeiner, 1993; Glipin et al., 1986; Melcangi et al., 2000; Russell et al., 1996; Yanai‐Inamura et al., 2019), which might cause bladder dysfunction symptoms if it compromises the LUT reflexes (Peng et al., 2008). Structural and biochemical changes of nerve fibers and the reduction of regenerative and reinnervating capabilities that come with age impairs the central and peripheral nervous system (Verdú et al., 2000), which might affect the LUT reflexes. Oshiro et al. (2021) showed external urethral sphincter atrophy in aged rats, and urethral sphincter muscle fibers appear to be replaced by connective tissue throughout aging (Yanai‐Inamura et al., 2019), both of which could lead to a more compliant urethra. Histological studies also showed a decline in number and density of striated urethral sphincter nerve and muscle fibers with age (Perucchini, DeLancey, Ashton‐Miller, Galecki, et al., 2002; Perucchini, DeLancey, Ashton‐Miller, Peschers, et al., 2002).

In this study, we hypothesized that afferent activity in the pudendal nerve, which carries urethral sensation, decreases with age, and explored how a combination of urethral afferent degradation and an increase in urethral compliance might contribute to a reduction in urethral sensory outflow. We recorded pudendal afferents (electroneurogram [ENG]) in young and old animals while we infused the urethra with fluid. We found that equivalent flow rates evoked less urethral afferent activity in the older rats. To understand what factors were driving the afferent reduction, we examined the pudendal nerves histologically and measured the pressures that developed in the urethra during infusion. We found evidence that axonal myelination decreased in old rats and lower pressures developed in the urethra of older rats during infusion.

## 2 METHODS

### 2.1 Ethical approval

All animal care and experimental procedures were reviewed and approved by the Institutional Animal Care and Use Committee at Florida International University. We used two groups of urethane‐anesthetized female Sprague–Dawley rats (young [3–7 months], old [18–24 months], *n* = 12 each group) to quantify the effect of aging on urethral afferent signaling. The age of old rats matches human lifespan in a reasonable way (Sengupta, 2013). All experiments were non‐survival. Rats were bought from Charles River Laboratories, and rats in the “old” group were kept for 6‐–12 months under low‐cholesterol diet (to keep them healthy) in an animal care facility under a 12:12‐h light‐dark cycle until they reached the appropriate age for the study. Animals were anesthetized initially with isoflurane gas followed by injection of 1.2 g/kg urethane (dissolved as 0.2 mg/ml in 0.9% saline solution) subcutaneously using a 27‐gauge needle. The foot pinch reflex of animals was tested 1 h after injection, and supplemental doses (0.1 g/kg, every 30 min) were injected until the foot withdrawal reflex abated. Urethane was used because it provides a stable depth of anesthesia and spares LUT reflexes (Matsuura & Downie, 2000). The body temperature and heart rate (determined via electrocardiogram) of animals were measured to monitor the vital signs and keep the depth of anesthesia. Animals were euthanized by an intraperitoneal injection of pentobarbital sodium after the experiment.

### 2.2 Experimental protocol

**FIGURE 1:**
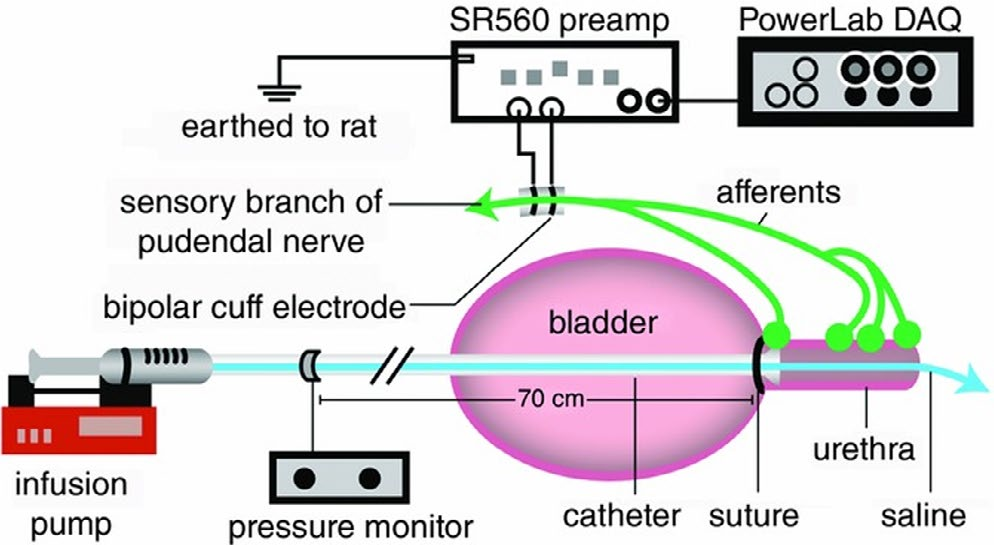
Experimental setup for nerve recording (Danziger & Grill, 2015). The catheter is inserted to the urethra through bladder dome and is tied with suture around bladder neck. The urethra is infused with selected flow rates and the neural activity of the pudendal afferents is recorded. The figure was taken from Danziger and Grill (2015) with permission

A catheter (PE90) was passed through the intravesical space into the urethra from the bladder dome to allow infusion of fluid through the urethra. The abdomen was sutured closed, but we left the bladder incision open so that the bladder remained empty during the experiment. A Millar pressure catheter (SPR‐1000; Millar) was passed through the urethral catheter and into the urethra just proximal to the trigon to measure the pressure in the proximal urethra. This low‐profile, solid state transducer let us record pressure while minimally interfering with flow. A computer‐controlled infusion pump (Ellite 11; Harvard Apparatus) was connected to the catheter and used to pass a solution of room temperature saline through the urethra at controlled flow rates. The pudendal nerve was exposed using the posterior approach and the sensory branch was isolated from the compound nerve and connective tissue. The sensory branch almost completely of sensory fibers, and can be isolated through careful dissection (Danziger & Grill, 2015; McKenna & Nadelhaft, 1986). A bipolar nerve cuff electrode (Platinum–iridium insulated with the contact spacing of 2 mm; World Precision Instruments) was placed on the sensory branch of the pudendal nerve to record the ENG. At 2 min intervals (the pudendal nerve sensory response to flow can recover after 2 min (Danziger & Grill, 2015)) the urethra was infused at a pseudorandomly selected flow rates ([0.1, 0.2, 0.3, 0.4, 0.5, 1, 2, 3, 5, 11] ml/min) for 15 s (this time is sufficient for the flow rates to develop required pressure in the urethra). Under physiological conditions, the average flow rate is volume voided divided by the duration of the voiding period, which is 6.4 ml/min in young rats) (Geramipour & Danziger, 2020). However, if we assume that urethral flow is only permitted during the silent phase (not the bursting phase), the average flow rate will be 10.4 ml/min (Geramipour & Danziger, 2020). If instead we assume that urethral flow is slowed during sphincter contractions, rather than stopped, then a typical average flow rate during voiding lies between 6.4 and 10.4 ml/min. We expect old rats to have lower flow rates on average during voiding and may also experience slow leaking from stress incontinence; therefore, the flow rates we used were chosen to represent a range from barely detectable to the maximum physiologically relevant flow the urethra might experience. Electrical activity of the pudendal sensory nerve was sampled at 20 kHz (PowerLab 8/35; ADInstruments), amplified at a gain of 10^4^, and band pass filtered from 10^2^ to 10^4^ Hz (SR560; Stanford Research Systems). Pressure signals were amplified at a gain of 10, sampled at 100 Hz and recorded unfiltered. The experimental setup is displayed in Figure 1.

The sensory branch of the pudendal nerve along with the cuff electrode was completely removed after the experiment, fixed in 10% buffered formalin, and embedded in paraffin. The middle segment of the nerve tissue was cut into slices with 2 μm thickness. We used hematoxylin and eosin staining (Hsieh et al., 2016), the light microscope (Leica DM 4500b) and ImageJ to measure axonal density and myelin thickness of the afferents in the sensory branch of pudendal nerve.

### 2.3 Data analysis

**FIGURE 2:**
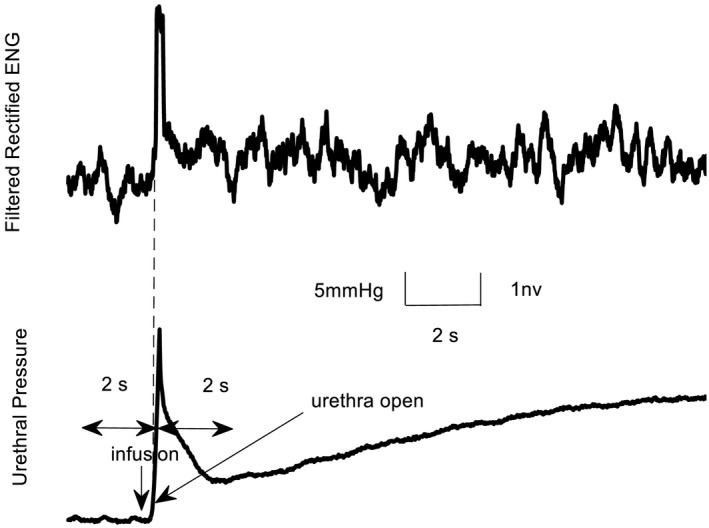
The initial transient response of pudendal nerve activity (ENG) and urethral pressure to flow. Urethral opening was defined as the time after flow onset (infusion) when the urethral pressure first exceeded the 75% of RMS value of the pressure across the entire duration of the flow. ENG, electroneurogram; RMS, root mean square

**FIGURE 3:**
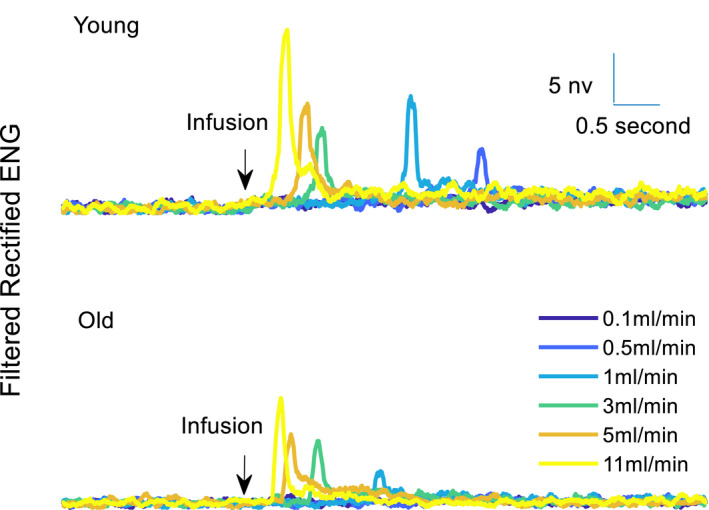
The transient response of pudendal afferent activity to a subset of the different applied flow rates in a typical young and old animal. Higher flow rates initiate more neural activity in both groups of animals. 0.5 ml/min urethral infusion evoked detectable levels of ENG in the young animal but could not generate activity in the old animal. ENG, electroneurogram

Before data analysis, ENG signals were rectified and filtered (100–3000 Hz). To better visualize the initial transient neural response to infusion, a moving average filter with a 200 ms sliding window was applied. A scalar measure of the initial transient peak in response to flow onset was used (Danziger & Grill, 2015) to quantify trends of the pudendal afferent activity and urethral pressure across flow rates and trials. To capture the transient response of urethral pressure to flow, we subtracted the root mean square (RMS) of baseline urethral pressure before urethral opening (caused by flow) from the RMS activity after urethral opening in a 2‐s window (Figure 2). Urethral opening was defined as the time after flow onset when the urethral pressure first exceeded the 75% of RMS value of the pressure across the entire duration of the flow. We subtracted the RMS of baseline neural activity before urethral opening (caused by flow) from the RMS activity after urethral opening in a 2‐s window to quantify change from baseline. We did not start the RMS calculation immediately (or at a fixed latency) after turning on the infusion pump because pudendal afferents are not activated until the urethra opens, and each flow rate required a different amount of time to overcome urethral opening pressure (Figure 3).

## 3 RESULTS

Pudendal sensory nerve activity was recorded in response to different urethral flow rates. Figure 3 shows the rectified and moving averaged filtered ENG response to flow in a young and old animal. These data show a large transient initial response to the onset of flow followed by persistent steady state activity. The ENG decayed back to baseline once the flow was terminated. The onset of neural activity with respect to the start of the infusion pump occurred earlier for high flow rates because higher flow rates require less time to develop enough catheter pressure to overcome the urethral opening resistance (Griffiths & Rollema, 1979). All flow rates except 0.1 ml/min could activate the pudendal afferents of young animals, probably because this flow rate is not strong enough to stretch the urethral wall sufficiently to activate the urethral afferents.

To capture the transient ENG response to flow onset, we subtracted the integrated RMS of baseline activity before urethral opening by flow from the RMS activity after urethral opening in a 2‐s window (see Figure 2). The same urethral flow rates evoked less nerve activity in older animals [(*F*(1,33) = 10.5, *p* = 0.003), mixed analysis of variance (ANOVA)] which shows there is a deficit in neural response to flow in older animals (Figure. 5).

Flow in the urethra increases the urethral pressure and distension, and the tension generated by the distension leads to activation of pudendal afferents (Danziger & Grill, 2015; Le Feber et al., 1998). However, the tension generated in the urethra caused by a fixed flow rate may be different across animals and across ages, which may affect the evoked neural activity in pudendal afferents. Urethral muscle diameter and elasticity are key factors that determine what pressure and tension will be generated in the urethra in response to fluid infusion. Therefore, maintaining constant infusion rates across animals and age groups does not guarantee we generate equal urethral pressures or tensions. Previous studies on mice (Liu et al., 2019), rat (Russell et al., 1996), and adult women (Chang et al., 2010) showed that the urethral diameter does not change with age, indicating that pressure and tension differences caused by equivalent flow rates are likely due to differences in compliance.

The precise transduction mechanism of mechanical stimuli into urethral afferent activity is not fully known (Danziger & Grill, 2016). However, due to lack of encapsulated sensory endings in the urethra (Johnson & Halata, 1991) it is likely that tension (rather than pressure) is the primary sensed stimuli; and given that afferent response to pressure is identical whether the fluid causing that pressure is flowing or static (Le Feber et al., 1998), it is likely that flow itself is not a primary transduction mechanism. Unfortunately, it is not feasible to measure circumferential urethral tension in vivo, and instead we measured urethral pressure as a surrogate. This is justified because tension is likely to be highly correlated with pressure under physiological conditions, meaning we would not expect qualitative differences in our results measuring tension rather than pressure (Le Feber et al., 1998, 2004). This is further supported by models that use functions of urethral pressure to accurately predict urethral afferent responses across a wide range of flow rates and flow profiles (Danziger & Grill, 2015; Le Feber et al., 1998).

**FIGURE 4:**
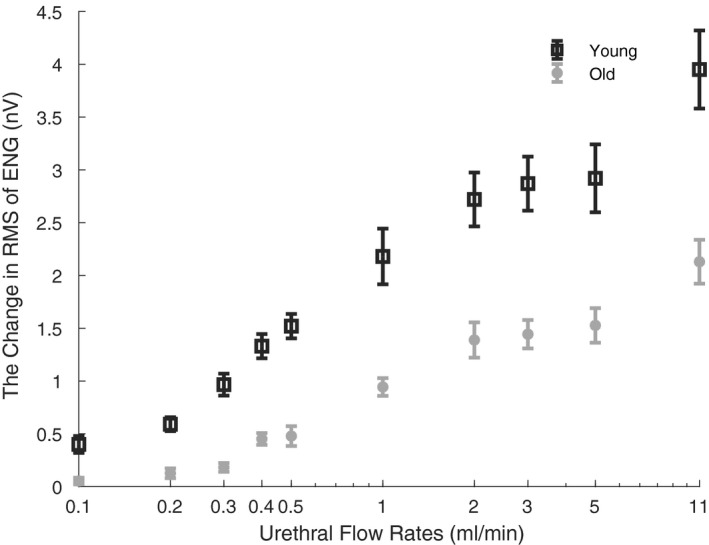
The initial transient peak response of pudendal nerve activity to urethral infusion in young (black) and old (gray) animals. The change in RMS of ENG response to all flow rates in a 2‐s window was computed and averaged across the corresponding age group. The same flow rates evoked less pudendal afferent activity in older animals. Each animal's average ENG response to multiple applications of each flow rate was computed, then that number was averaged across all animals in each age group for each flow rate. Bars are standard error across animals (*n* = 12 per age group), plotted on a log‐scale horizontal axis. ENG, electroneurogram; RMS, root mean square

By computing the pressure evoked by each applied flow rate, we found that the same flow rates evoked less pressure in the urethra in older animals than younger ones [*F*(1,33) = 8.7, *p* = 0.006, mixed ANOVA, Figure 4]. The change in urethral pressure was computed by subtracting the integrated RMS of baseline pressure before urethral opening by flow from the RMS activity after urethral opening in a‐2 s window (see Section 2.3). The likely explanation for this difference in pressure between age groups is that, the urethral lumen in old rats is more compliant and thus experiences less pressure at equivalent flow rates.

**FIGURE 5:**
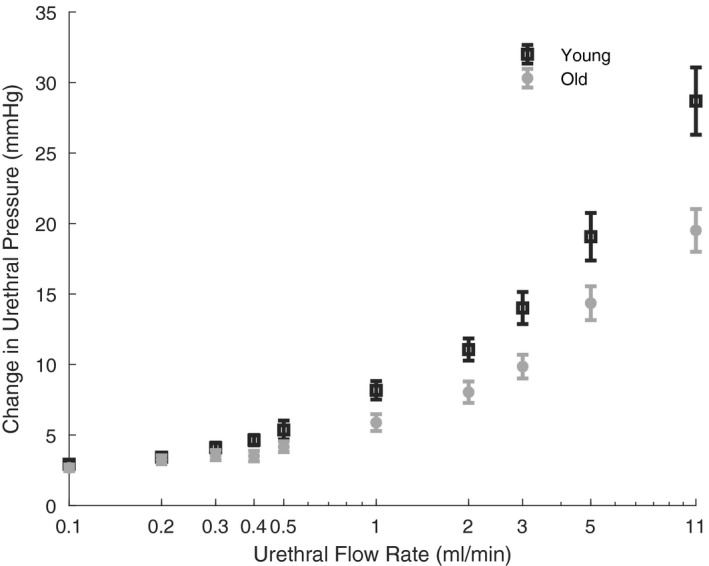
The flow‐evoked urethral pressure response in young and old animals. The change in RMS of urethral pressure was computed in a 2‐s window for each flow rate and averaged across the corresponding animal age groups. The same urethral flow rates created less urethral pressure in older animals. The change in urethral pressure is displayed as the mean with standard error, plotted on a log‐scale horizontal axis. RMS, root mean square

**FIGURE 6:**
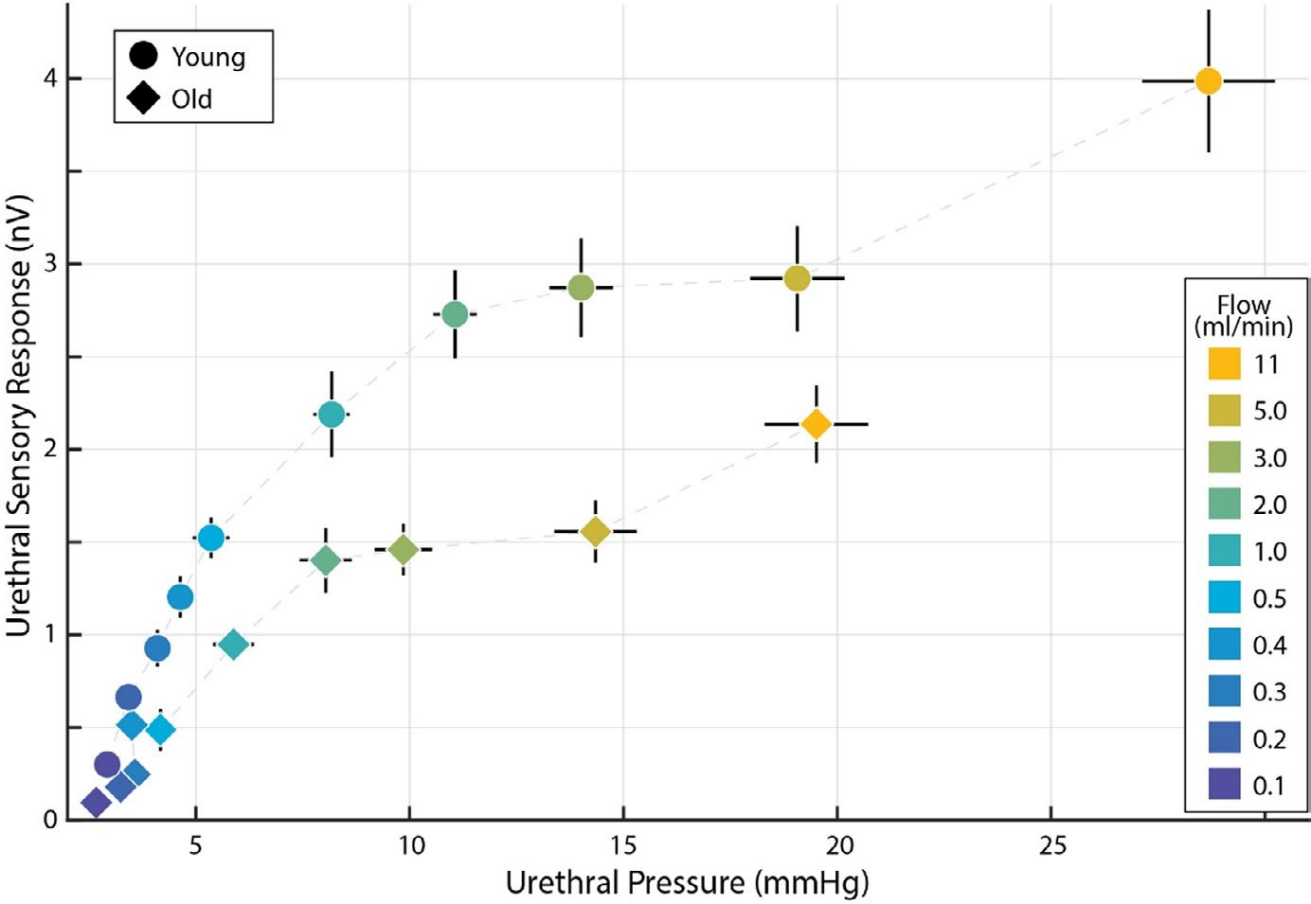
Old rats (diamonds) have smaller urethral sensory responses than young rats (circles) to equivalent pressures generated by experimentally applied flow rates (color). Larger flow rates were required in old rats to generate the same urethral pressures (see also Figure 4), but even when comparing old and young rats at equivalent pressures (the same extent along the abscissa) old rats showed less sensory response. Ordinate data were pooled according to Figure 5 and abscissa data were pooled according to Figure 4, error bars for both are standard error

The lower pressures in older animals may partially explain their lower flow‐evoked ENG response (Figure 5). To understand the relationship between urethral pressure and evoked afferent activity, we averaged the resulting urethral pressures and averaged the evoked afferent activity for each applied flowrate across each age group. This let us visualize the relationship between pressure (dependent variable) and evoked ENG (dependent variable) across each flow rate (color–independent variable) for both young (circles) and old (diamonds) rats. Figure 6 shows that evoked ENG in old rats is strictly less than for young rats at equivalent urethral pressures, which controls for the effects of differential tissue‐related responses to fluid infusion between old and young rats. For example, the pressure evoked by the 5 ml/min infusion in young animals is approximately equal to the pressure evoked by 11 ml/min in old animals (approximately 19 mmHg in both cases); however, the ENG response to 11 ml/min in old group is less than the ENG response to 5 ml/min in young group. Therefore, an increase in passive urethral compliance with age, which results in less intraurethral pressure compared to young rats, is not sufficient to explain the entirety of the reduction in urethral afferent signaling we observe in old rats.

**FIGURE 7:**
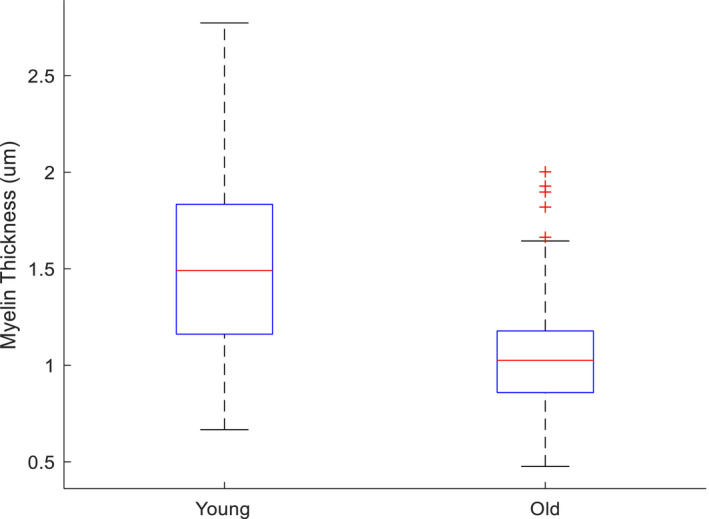
Axonal myelin in the sensory branch of the pudendal nerve is thicker in younger animals

**FIGURE 8:**
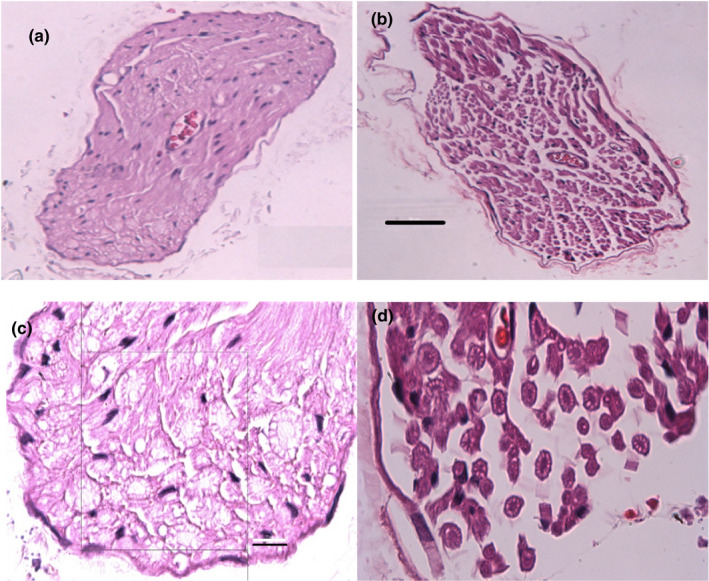
The examples of axons in the sensory branch of the pudendal nerve in young and old animals. (a) (young), (b) (old) are with 20× magnification and (c) (young), (d) (old) are with 100× magnification. The black bar in (b), shows 50 μm in (a) and (b); and the black bar in (c), shows 10 μm in (c, d)

To examine the nerve‐related factors that could lead to reduced afferent signaling we measured the axonal density (three young and three old), and myelin thickness (six young and six old) of the sensory branch of the pudendal nerve after the experiments. The myelin thickness (young group: 1.53 ± 0.03 μm; elderly group: 1.03 ± 0.01 μm; *p* < 0.001, *t*‐test) of the elderly group was smaller than those of the young group. The axonal density of the young and old group was 0.013 ± 0.004 and 0.01 ± 0.006 fiber/µm^2^, respectively. Figure 7 shows the boxplot of pooled myelin thickness from all samples in young and old animals. Figure 8a,b shows the examples of axons in young (Figure 8a) and old (Figure 8b) animals with 20x magnification and Figure 8c,d shows the examples of axons in young (Figure 8c) and old (Figure 8d) animals with 100× magnification.

## 4 DISCUSSION

We measured age‐related loss of urethral signaling capability by measuring the pudendal afferent activity directly. We found that urethral afferents (measured by ENG) are less responsive to fluid flow in old rats. We also found that pressure in the urethra decreases with age, meaning their lumen experiences less pressure than for the same flow rate in the urethra of young rats. We showed that even after controlling for age‐related pressure differences, less activity is generated in pudendal afferents of old animals at equivalent pressures. Histological results of the pudendal nerve showed significant myelin thickness reduction with age, suggesting the nonmechanical factors have a neuropathic origin. Our results suggest that this age‐related reduction in urethral afferent outflow is mediated by a mechanical component, presumably a more compliant urethra, and a nonmechanical component, presumably neuropathy.

There are several possible explanations for less flow‐evoked pudendal afferent activation in old animals. The horizontal axis in Figure 6 indicates that with the same urethral flow rate, less pressure (a correlate of wall tension) is generated in the urethra of older animals, suggesting that mechanical changes in the urethral tissue attenuates the physical stimulus that urethral afferents respond to. Interestingly, even at the same urethral pressure, less neural activity is generated in the pudendal afferents of old animals. Therefore, an increase in passive urethral compliance with age, which results less intraurethral pressure compared to young rats, is not sufficient to explain the entirety of the reduction in urethral afferent signaling we observe in old rats.

### 4.1 Computing the contributions of mechanical and nonmechanical factors to total sensory loss

**FIGURE 9:**
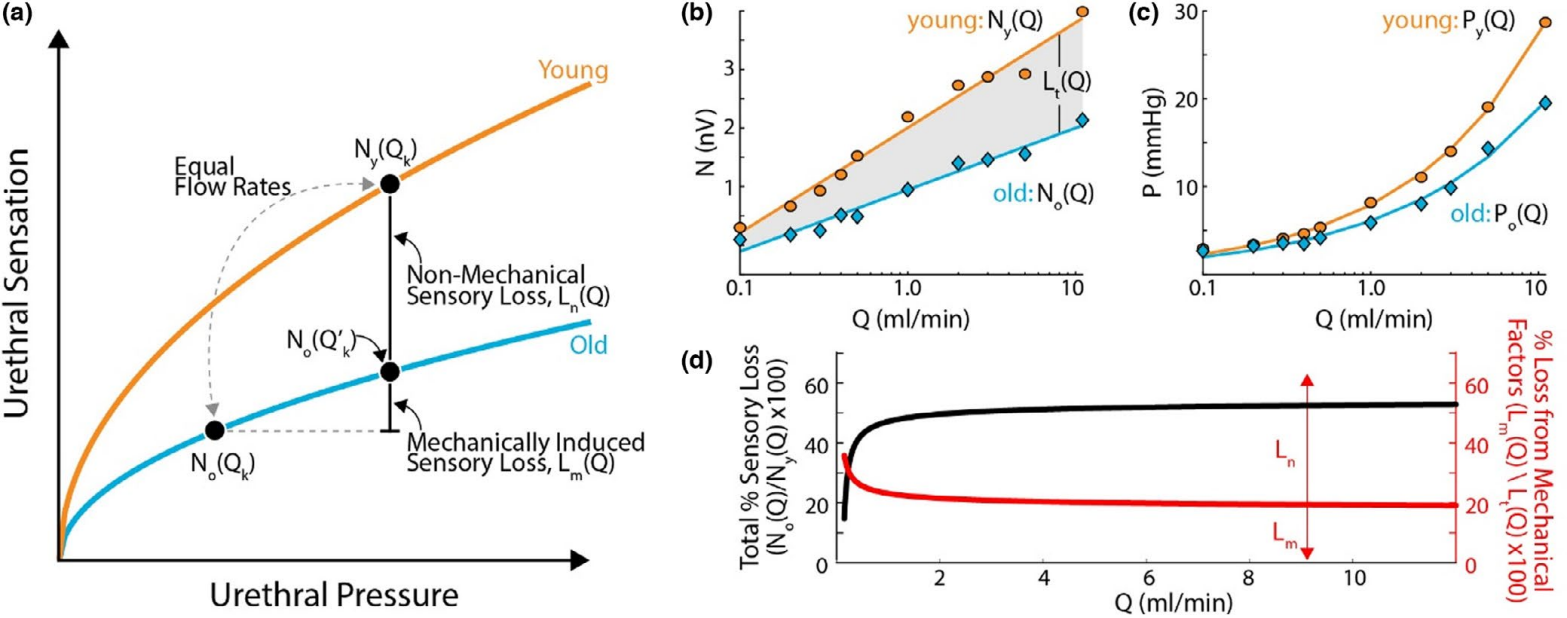
Decomposition of age‐related urethral sensory loss into its mechanical and nonmechanical factors. (a) Schematic (not actual data) illustrating how the total sensory loss in old rats breaks down into its nonmechanical and mechanical components. The first equality in Equation (1) expresses this depiction of the total loss. (b) Curve fits to data in Figure 5 for neural response as a function of applied flow rate. The gray patch depicts the total sensory loss, expressed in the second equality in Equation (1). (c) Curve fits to data in Figure 4 expressing urethral pressure as a function of applied flow rate. (d) The ratio of evoked neural activity in old to young rats as a function of applied flow rate (black). The percentage of sensory loss in old rats due to mechanical factors as a function of applied flow rate

Here, we quantify the contributions to pudendal afferent loss of sensitivity from mechanical factors, which change the pressure and tension the urethra experiences in response to flow, and nonmechanical factors, which are putatively intrinsic to the nerve itself. We find that old rats lose over 50% of their urethral sensitivity overall when compared to young rats, and that 20% of that loss is due to mechanical factors of the urethra, with the remaining 80% from intrinsic neural factors (Figure 9d). The remainder of this subsection describes the procedure to obtain this result.

The total loss of urethral sensitivity is the difference in evoked afferent responses between old and young rats to the same flowrate (quantified in Figure 4). To visualize how this total decomposes into the two factors, in Figure 9a we show a schematic representation of the afferent response to flow in the urethra as a function of pressure (actual data are in Figure 6). Formally,

(1) $$L_{t}\left(Q\right)=L_{n}\left(Q\right)+L_{m}\left(Q\right)=N_{y}\left(Q\right)‐N_{o}\left(Q\right)$$

where *L*_t_(*Q*) is the total loss as a function of flow rate, *Q*, and *N*_y_(*Q*) and *N*_o_(*Q*) are the neural responses of young and old rats as a function of flow rate. The first equality in Equation (1) states that the total sensory loss is the sum of the sensory loss due to nonmechanical (or neural) factors, *L*_n_(*Q*), and mechanical factors, *L*_m_(*Q*), which is shown in Figure 9a. The second equality is visualized as the gray patch in Figure 9b, which is the difference between nerve responses of young and old animals as a function of flow rate.

At a given urethral pressure, the difference between the neural afferents of young and old rats must be the nonmechanical contribution to sensory loss, since the mechanical contribution only has its effect through changes in the pressures (and therefore tension) the urethra experiences. This is shown as the difference between the young and old curves in Figure 9a. We can write this as

(2) $$L_{n}=N_{y}\left(Q_{k}\right)‐N_{o}\left(Q_{k}^{\prime }\right)$$

where *N*_y_(*Q*_k_) is the neural response of the young rats to flow rate *Q*_k_, and $N_{o}\left(Q_{k}^{\prime }\right)$ is the neural response of the old rats at flow rate $Q_{k}^{\prime }$, which is the flow rate required to generate urethral pressure in old rats that *Q*_k_ generates for young rats. The remaining mechanical contribution to sensory loss is then the difference between the neural response of old rats at the given pressure minus the sensory response of the old rats to the flow rate that produced the given pressure in young rats (shown geometrically in Figure 9a):

(3) $$L_{m}=N_{o}\left(Q_{k}^{\prime }\right)‐N_{o}\left(Q_{k}\right)$$

To compute *L*_n_ and *L*_m_ we need expressions for *N*_y_(*Q*), *N*_o_(*Q*), and $Q_{k}^{\prime }$. *N*_y_(*Q*) and *N*_o_(*Q*) can be computed by direct curve fits to data in Figure 5 using the form *N*(*Q*) = *c*_1_ + *c*_2_log_10_(*Q*), yielding the fits shown in Figure 9b. The constants *c*_1_ and *c*_2_ were fit separately for each age group (*c*_1y_ = 2.00, *c*_2y_ = 1.79, *c*_1o_ = 0.94, *c*_2o_ = 1.05), and yield *R* ^2^ = 0.98 and *R* ^2^ = 0.97 for young and old groups. To compute $Q_{k}^{\prime }$ (the flow rate in old rats that causes the same pressure as the flow rate *Q*_k_ causes in young rats) we require an expression relating the pressure to flow for young rats, *P*_y_(*Q*), and old rats *P*_o_(*Q*). We can obtain these expressions through direct curve fits to data in Figure 4 using the form $P\left(Q\right)=b_{1}Q^{b_{2}}$, yielding the fits shown in Figure 9c. The constants $b_{1}$and $b_{2}$were fit separately for each age group (*b*_1y_ = 7.90, *c*_2y_ = 0.54, *c*_1o_ = 6.08, *c*_2o_ = 0.49), and yield *R* ^2^ = 0.99 and *R* ^2^ = 0.99 for young and old groups. To compute *Q*′ in general, we set *P*_y_(*Q*) = *P*_o_(*Q*′), which yields $Q^{\prime }={\left(\frac{b_{1y}}{b_{1o}}Q^{b_{2y}}\right)}^{1/b_{2o}}$. Plugging *Q*′ into Equation (3), and using the expressions for *N*_y_(*Q*) and *N*_o_(*Q*), we can decompose the loss into the mechanical and nonmechanical factors. The mechanical loss as a function of flow rate is then

(4) $$L_{m}\left(Q\right)=c_{2o}\log_{10}\left({\left(\frac{b_{1y}}{b_{1o}}\right)}^{1/b_{2o}}Q^{\frac{b_{2y}}{b_{2o}}‐1}\right)$$

The red trace in Figure 9d shows the percentage of the total loss, *L*_t_(*Q*), due to mechanical factors, *L*_m_(*Q*), expressed as *L*_m_(*Q*)/*L*_t_(*Q*) · 100. For the larger flow rates that occur during voiding, mechanical factors account for only 20% of the total sensory loss, with the balance coming from nonmechanical factors. Since the mechanical loss is proportional to log of $Q^{\frac{b_{2y}}{b_{2o}}‐1}$, and the exponent is <1, we expect a very weak dependence of *L*_m_(*Q*) on flow rate for flows above 1 ml/min. Mechanical factors may represent a larger share of the impact at low flow rates, as voiding is beginning, although at low flow rates the total sensory loss is much lower overall. The black trace in Figure 9d shows the overall percentage of neural activation that old rats lose, *N*_o_(*Q*)/*N*_y_(*Q*) · 100, which indicates most of the functional loss comes at high flow rates. Taken together, most of the age‐related urethral sensitivity loss occurs at higher flow rates, and at higher flow rates over 80% of the loss originates with nonmechanical factors, which suggests that urethral afferents may be a promising therapeutic target.

### 4.2 Drivers of urethral sensation loss

One potential explanation for the observed decrease in pudendal afferent activity could be age‐related degradation of the urethral musculature (i.e., a mechanical factor). If the urethra is more compliant (very loose), the flow cannot distend the urethra to develop enough tension and activate pudendal afferents. Since urethral tension is the putative driver of urethral afferents, rather than flow (Le Feber et al., 1998), this might also decrease the activity of pudendal afferents. Our results showed that the same flow rates evoked less pressure (correlated with tension) in the urethra in older animals. The increased external urethral sphincter (EUS) connective tissue, reduced number of EUS fibers (Yanai‐Inamura et al., 2019) and EUS muscle atrophy (Oshiro et al., 2021) with age might explain the reduced pressure generated by urethral infusion in older animals.

Deficits in flow‐evoked pudendal nerve activity may be the consequence of structural and biochemical changes that result in a slowly progressive loss of neurons and myelin (i.e., a neural factor). Therefore, age‐related degradation of the pudendal nerve might be another contributing factor for less flow‐evoked ENG response in older animals. The myelin thickness of the old group was significantly smaller than those of the young group. Previous research has revealed significant reduction in mRNA and protein levels for myelin sheath components in the sciatic nerves of older rats (Melcangi et al., 2000). A decrease in myelin of the myelinated fibers in the aged subjects is the main morphologic change responsible for the nerve conduction changes and this delay might change the normal timing of neural circuit in the spinal cord and disrupt LUT reflexes (Geramipour & Danziger, 2020).

Reduced number of urethral cells expressing serotonin (5‐hydroxytryptamine [5‐HT]) with age can be another contributing factor for less pudendal afferents activity in older animals (Coelho et al., 2019). It is believed that cross‐talk between urethral afferents and urethral cells expressing serotonin involved in transmission of sensory information from the urethra to the central nervous system (Kullmann et al., 2018). Therefore, the lower number of 5‐HT‐positive urethral cells may be responsible for decreased excitation of the urethral afferents (Coelho et al., 2019).

### 4.3 Study limitations

The methodology of our study has a number of limitations that we note here to help clarify the interpretation of our results. (1) We tied a catheter around the bladder neck to infuse the urethra. This might prevent mechanical distension of the proximal urethra and influence flow‐evoked pressure (or tension) and afferent activity results. (2) It is possible that urethane anesthesia dosed according to body weight (the gold‐standard approach) does not result in equal responses in young and old animals. If urethane suppresses afferent activity or urethral muscle tone to a different extent in old rats than young rats, the anesthesia itself could contribute to the differences we observe between ages. (3) Our study isolated and quantified the fundamental response of urethral afferents and urethral pressure to applied urethra flow. To perform this characterization, we needed to remove many potentially confounding aspects present in natural voiding, which included enforcing a constant flow rate throughout urethral infusion and maintaining an empty bladder, neither of which is typical of physiological voiding. Therefore, our results specifically highlight changes in fundamental afferent activity related to aging and cannot be used to directly infer functional age‐related differences. This work only discovered that age‐related differences in afferent responses and pressures exist, but their explanatory role in functional age‐related LUT decline remains to be determined. (4) We measured urethral pressure rather than tension, which is very likely to the primary sensed urethral stimuli. If these two quantities diverge substantially during infusion, our estimate of the mechanical effects on age‐related afferent loss may also change. We expect any effects of using pressure as a surrogate for tension to be small, since these variables will be highly correlated with each other in many physiological conditions, and multiple models have shown success predicting urethral afferent discharge from pressure information.

### 4.4 Possible connections with underactive bladder: future directions

Underactive bladder (UAB) is very common in elderly, and its etiology is under studied (Chuang et al., 2015); here we propose a mechanism that could explain age‐related UAB symptoms that is consistent with the literature and the results of this work. In prior work we showed that there is a deficit in the urethra‐to‐bladder (augmenting) reflex that worsens with age (Geramipour & Danziger, 2020). Disrupting the augmenting reflex by administration of intraurethral lidocaine (Geramipour & Danziger, 2020; Jung et al., 1999; Shafik et al., 2003), or transecting the pudendal nerve (Peng et al., 2008) significantly decreases voiding efficiency, which is a hallmark UAB symptom. Here we report that the urethral afferents, the fibers directly responsible for activating the augmenting reflex, weaken with age. The afferent weakening we observed is consistent with the reported decline in urethral sensation and neuropathy in age‐related UAB (Fidas et al., 1987; Parys et al., 1988). We hypothesize that age‐related neuropathy and increases in urethral compliance lead to less urethral sensory outflow that, in turn, leads to a less sensitive and robust augmenting reflex. Since the augmenting reflex is necessary for efficient voiding, a neuropathic weakening of this reflex could contribute to the poor voiding efficiency we observe in age‐related UAB patients. To fully verify this hypothesis, further work is needed to demonstrate (1) that the reduction in urethral afferent outflow is causally responsible for the weakened augmenting reflex in old rats, and (2) that a weakened augmenting reflex materially contributes to the loss of voiding efficiency or hesitancy observed in UAB.

## CONFLICT OF INTEREST

No conflict of interest, financial, or otherwise are declared by the author(s).

## Data Availability

Data is available upon request.
